# 

**Published:** 1908-05-02

**Authors:** 


					HOSPITAL
Telegraphic Address i THE MODERN Telephone Number :
Ospodole, London. |[ EPICAL. JOURNAL. ^
?jf-il U?v,y>;\rr"i?l ?-
"EW SERIES. No. 59,
Nn 1131. Vol Yl IV. OATimiJAYv--
T A/A.V !I ? ?. .
*" - . * * ? <l'-X <
\^90g. r
* >PRICE THREEPENCE.

				

